# Characterization of a Novel Hemolytic Activity of Human IgG Fractions Arising from Diversity in Protein and Oligosaccharide Components

**DOI:** 10.1371/journal.pone.0085711

**Published:** 2014-01-21

**Authors:** Shaoying Min, Fang Yan, Yueling Zhang, Xiangqun Ye, Mingqi Zhong, Jinsong Cao, Haiying Zou, Jiehui Chen

**Affiliations:** 1 Department of Biology and Guangdong Provincial Key Laboratory of Marine Biotechnology, Shantou University, Shantou, China; 2 Mariculture Institute of Shandong Province, Qingdao, China; 3 Medical College, Shantou University, Shantou, China; Universidade Federal de Minas Gerais, Brazil

## Abstract

Human IgG is a well-established multifunctional antigen specific immunoglobulin molecule of the adaptive immune system. However, an antigen nonspecific immunological function of human IgG has never been reported. In this study, human IgG was isolated using ammonium sulfate fractional precipitation and diethylaminoethanol (DEAE) cellulose 52 ion exchange chromatography, from which h-IgG and hs-IgG fractions were purified on the basis of their differential binding to rabbit anti-shrimp hemocyanin antibody (h) and rabbit anti-shrimp hemocyanin's small subunit antibody (hs), respectively. We found that h-IgG had a higher hemolytic activity than hs-IgG against erythrocytes from humans, rabbits, mice and chickens, whereas the control IgG showed negligible activity. h-IgG could interact directly with erythrocyte membranes, and this interaction was suppressed by high molecular weight osmoprotectants, showing that it may follow a colloid-osmotic mechanism. In comparative proteomics and glycomics studies, h-IgG and hs-IgG yielded 20 and 5 significantly altered protein spots, respectively, on a 2-D gel. The mean carbohydrate content of h-IgG and hs-IgG was approximately 3.6- and 2-fold higher than that of IgG, respectively, and the α-d-mannose/α-d-glucose content was in the order of h-IgG>hs-IgG>IgG. In this study, a novel antigen nonspecific immune property of human IgG was investigated, and the diversity in the protein constituents and glycosylation levels may have functional signficance.

## Introduction

Immunoglobulins consist of four polypeptides, namely, two heavy and two light chains, each of which contains a variable region at the amino terminus and a constant region at the carboxyl terminus [1], [2]. Five human immunoglobulin classes, namely IgM, IgG, IgA, IgE and IgD, have been identified in blood. Among them, IgG is the most abundant, constituting over 75% of the immunoglobulins in serum. IgG plays a primary role amongst immunoglobulins in immune defense. Moreover, human IgG is multifunctional, with an involvement in neutralization of toxins and viruses, agglutination and precipitation, complement binding and activation, binding to macrophage Fc-receptors and antigen-dependent cellular cytotoxicity [3]–[5].

It is generally accepted that human IgG only possesses antigen specific immunological functions. Interestingly, recent evidence indicates that human IgG and a large variety of antigen nonspecific immunological molecules such as rabbit C-reactive protein (CRP) [6], *Lymnea stagnalis* molluscan defence molecule [7], *Hyalophora cecropia* hemolin [8], *Aplysia californica* aplysia cell adhesion molecule (apCAM) [9], *Drosophila* Down syndrome cell adhesion molecule (Dscam) [10], *Branchiostoma floridae* variable region-containing chitin-binding proteins (VCBPs) [11] and *Biomphalaria glabrata* fibrinogen-related proteins (FREPs) [12] may share a common evolutionary origin. In particular, our previous research found that human IgG not only contained several conserved regions with shrimp hemocyanin, but could also cross-react with rabbit anti-shrimp hemocyanin antibodies [13], [14]. In addition, hemocyanin in mollusks and arthropods has been documented to possess several immune functions, such as phenoloxidase [15], antiviral [16], antibacterial [17], antitumoral [18], hemolytic [19] and hemagglutinative [14] activities. Therefore, it is of great interest to further investigate whether human IgG also possesses some antigen nonspecific immune properties.

In the present study, two human IgG fractions were purified using affinity chromatography, and these molecules showed hemolytic activity, which was likely associated with the diversity in amino acid sequence and glycosylation of the IgG fractions. The results will contribute to the investigation of the evolutionary relationships between the adaptive immune molecules in vertebrates and the innate immune molecules in invertebrates.

## Materials and Methods

### Purification and identification of human IgG fractions

Human blood was harvested from 50 healthy individuals in the First Affiliated Hospital of Shantou University, Shantou, Guangdong Province, China. The study protocol was approved by the Institutional Animal Care and Use Committee of Shantou University. Written informed consent was also obtained from the healthy individuals before the start of the study. Serum was collected by centrifugation at 3,000 *g* for 20 min, pooled and stored at −20°C until needed. Human IgG fractions were isolated using ammonium sulfate fractional precipitation, diethylaminoethanol (DEAE) cellulose 52 ion exchange chromatography and affinity chromatography as described previously, with modifications [14]. Briefly, IgG was precipitated with ammonium sulfate at room temperature, and fractions between 33–50% saturation were collected by centrifugation at 12,000 g for 10 min at 4°C. The pellet was redissolved in a minimum volume of 0.01 M phosphate-buffered saline (PBS; pH 7.4) and subjected to extensive dialysis against the buffer overnight. The dialyzed fraction was purified using DEAE cellulose 52 ion exchange chromatography. The first peak was collected and used as the control IgG, which was then further purified by affinity chromatography. Two affinity chromatography columns were used, one containing rabbit anti-shrimp hemocyanin antibody (h), and the other containing the rabbit anti-shrimp hemocyanin's small subunit antibody (hs). DEAE-purified IgG (400 µl) was loaded onto each affinity column. After incubation for 3 h at room temperature, the columns were washed with 0.01 M PBS (pH 7.4) and eluted with 0.1 M glycine-HCl buffer (pH 2.4). Both unbound IgG fractions (named as unh-IgG and unhs-IgG) from the wash fractions and bound IgG fractions (named as h-IgG and hs-IgG) from the eluted fractions were collected and subjected to further analysis. Protein concentration was determined using the Bradford assay [20]. Unbound and bound IgG fractions were characterized using sodium dodecyl sulfate polyacrylamide gel electrophoresis (SDS-PAGE) under reducing conditions (3% stacking gel, 10% separating gel). For immunoblotting assays, proteins were transferred onto a polyvinylidene fluoride (PVDF) membrane with a semi-dry transfer apparatus according to the manufacturer's instructions. The membrane was blocked for 1 h with 5% skimmed milk in Tris-buffered saline (TBS; 20 mM Tris, 0.15 M NaCl, pH 7.4) at room temperature, then incubated with rabbit anti-human IgG antisera (1∶1,000 dilution) and goat anti-rabbit IgG-horseradish peroxidase (HRP; 1∶2,000 dilution) antibodies at room temperature for 1 h. Finally, the membrane was washed and developed with substrate 3,3′-diaminobenzidine (DAB) until optimum color was developed.

### Preparation of erythrocyte suspensions

Mature mice (CD1 strain), White New Zealand rabbits and White Leghorn chickens were housed in a temperature- and light-controlled environment with free access to regular food and water. Blood was harvested from the healthy humans, mice, rabbits and chickens. Of these, human blood was acquired from the same healthy individuals as described in the above section on the purification and identification of human IgG fractions. All animal procedures were approved by the Institutional Animal Care and Use Committee of Shantou University. The collected blood was centrifuged at 2,000 g for 10 min to obtain erythrocytes. Cells were washed three times with 0.01 M PBS (pH 7.4) and centrifuged at 500 g for 5 min. Erythrocytes were then diluted with 0.01 M PBS (pH 6.0) containing 0.15 M NaCl and 10 mM CaCl_2_ to obtain a 0.5% (v/v) suspension.

### Determination of hemolytic activity

Hemolytic activity was determined as previously described [21]. In brief, each IgG fraction (0.9 ml, 0.01 mg/ml) was mixed with 0.5% (v/v) erythrocyte suspension (0.3 ml). After incubation at 37°C for 1 h, unbroken cells and cell debris were removed by centrifugation at 3,500 g for 10 min, and hemolysis was determined by measuring the absorbance at 540 nm in supernatants. Next, 0.5% (v/v) erythrocyte suspensions were treated with double distilled water (ddH_2_O) or 0.01 M PBS-Ca^2+^ (pH 6.0) and used as 100% and 0% hemolysis controls, respectively. All samples were prepared in triplicate. The percentage of hemolysis was calculated as (A - A_0_)/(A_100%_ - A_0_)×100%. To further investigate the process of IgG-dependent hemolysis, kinetic analysis of chicken erythrocyte hemolysis was performed. A 0.5% (v/v) chicken erythrocyte suspension (10 µl) was mixed with 5 µg/ml h-IgG (10 µl) on a slide. After incubation at 37°C for 15, 30, 45 and 60 min, digital photomicrographs were taken with an Olympus BH-2 microscope (Olympus Company,Tokyo, Japan).

### Interaction of IgG fractions with erythrocyte membranes during hemolysis

To further confirm whether the human IgG fractions are involved in hemolysis, SDS-PAGE and immunoblotting were performed as described by Promdonkoy and Ellar with modification [22]. Briefly, A 0.5% (v/v) erythrocyte suspension (0.3 ml) was incubated with 0.1 mg/ml h-IgG (0.9 ml) at 37°C for 1 h, and erythrocyte membranes were collected by centrifugation at 3,500 g for 10 min and washed twice with 0.01 M PBS (pH 7.4) buffer. Erythrocyte membrane pellets were solubilized in 2 X protein loading buffer at room temperature before SDS-PAGE (3% stacking gel, 10% separating gel). A suspension of erythrocytes incubated with ddH_2_O was used as a control. The subsequent immunoblotting analysis was carried out as described above for the identification of human IgG fractions. Following the first SDS-PAGE, the band binding to anti-human IgG specifically around 121 kDa was further purified as Kang and Tong described [23]. In brief, the first SDS-PAGE gel was stained by Coomassie Brilliant Blue fast staining reagent (45% ethanol, 10% acetic acid, 1 mg/ml Coomassie Brilliant Blue R-250) for 20 min and then destained by 250 mM KCl. The band was excised and transferred into a dialysis bag. After electrophoresis in the SDS-PAGE electrode buffer overnight at 4°C, the eluate in the bag was concentrated by precooling acetone at −20°C for 30 min, then solubilized in protein loading buffer and heated for 5 min. And the following procedures including the second SDS-PAGE and immunoblotting were same as above described.

### Osmotic protection assay

The osmotic protection assay was performed as described [24] with modifications. Chicken erythrocytes (0.5%, v/v) were suspended in 0.01 M PBS (pH 6.0) containing 0.15 M NaCl, 0.01 M CaCl_2_ and one of the following osmoprotectants: 0.015 M polyethylene glycol (PEG) 4000, 6000 and 8000. Hemolysis was initiated by the addition of 0.9 ml h-IgG (0.01 mg/ml) to a 0.3 ml erythrocyte suspension with or without an osmoprotectant. The hemolysis was assessed as described above.

### Two-dimensional gel electrophoresis (2-DE)

We performed 2-DE of the IgG fractions as described previously [25]. A total of 20 µg of IgG, h-IgG or hs-IgG in rehydration buffer (7 M urea, 2 M thiourea, 4% CHAPS, 0.2% DTT and 3.4 µl of immobilized pH gradient [IPG] buffer, pH 3–10) was used to rehydrate the IPG strip (7 cm, pH 7–10, Bio-Rad, USA) for 16 h. Isoelectric focusing (IEF) was performed at a constant temperature of 20°C using a continuous increase in voltage (up to 4000 V) for 65,000 Vh. Prior to the second dimension, the IPG strip was incubated for 15 min in equilibration buffer (20% [w/v] glycerol, 2% SDS, 2% DTT, 0.375 M Tris-HCl, pH 8.8) then further equilibrated for 15 min in equilibration buffer containing 2.5% iodoacetamide instead of 2% DTT. The strip was placed onto a 10% SDS-PAGE gel. Molecular weight markers were loaded onto a filter paper and placed next to the IPG strip. Low-melting point agarose was used to cover the IPG strip and filter paper. Proteins were separated using the same conditions described above for SDS-PAGE analysis of human IgG fractions.

### Imaging analysis

The 2-DE gel images were analyzed with PDQuest software version 8.0 (Bio-Rad, CA). Comparative analysis of protein spots was performed by matching corresponding spots across different gels. Each of the matched protein spots was rechecked manually. The intensity of individual spots was normalized against the total intensity of all spots present in each gel, and subjected to statistical analysis to compare the normalized intensity of individual h-IgG or hs-IgG spots to those of control IgG. Significantly altered proteins were chosen using the following criteria: (1) *P* values<0.05, (2) means of both groups in the unpaired Student's *t*-test were increased or decreased >2.0-fold and (3) the change was consistent in all replicates for each group of IgG fractions.

### Sequence alignments of human IgG and hemolysins

For the prediction of potential specific amino acid sequences related to the hemolytic properties in human IgG, the amino acid sequences of human IgG heavy chain (AAA02914) and three hemolysins of *Serratia marcescens*, *Edwardsiella tarda* and *Xenorhabdus bovienii* from the NCBI database were aligned using sequence alignment programs (Clustal X and BioEdit).

### Determination of the carbohydrate content of human IgG fractions

The carbohydrate content was determined by the colorimetric method [26]. Glucose (0.01 mg/ml) was serially diluted with distillated water to a final volume of 2.0 ml, and 1.0 ml phenol (6% m/v) and 5.0 ml sulfuric acid were added immediately. After standing at room temperature for 25 min, the absorbance was measured at 490 nm, and the average of duplicate samples was used to plot a standard curve.

The α-d-mannose/α-d-glucose content was further measured using dot-blotting for lectin method [27]. Nitrocellulose (NC) membranes were cut into the desired size, soaked in TBS (20 mM Tris, 150 mM NaCl, pH 7.4) for 30 min and dried on a filter paper. Different concentrations of human IgG fractions were prepared (0.3 and 0.15 mg/ml), and 2-µl aliquots of each concentration were spotted onto the NC membrane. After drying, the NC membrane was blocked at room temperature for 2 h with 2% polyvinylpyrrolidone (PVP) 360,000 in TBS, washed three times with TBS for 10 min each time and reacted with a 1∶10 dilution of biotinylated concanavalin A (ConA) and with a 1∶1,000 dilution of avidin-peroxidase for 2 h at room temperature. The membrane was washed with TBS for 10 min a further three times, then reacted with DAB until optimum color was developed. The map of the precipitation dots was scanned and analyzed by the analytic system of GDS8000PC.

## Results

### Isolation of two human IgG fractions

**Figure 1:**
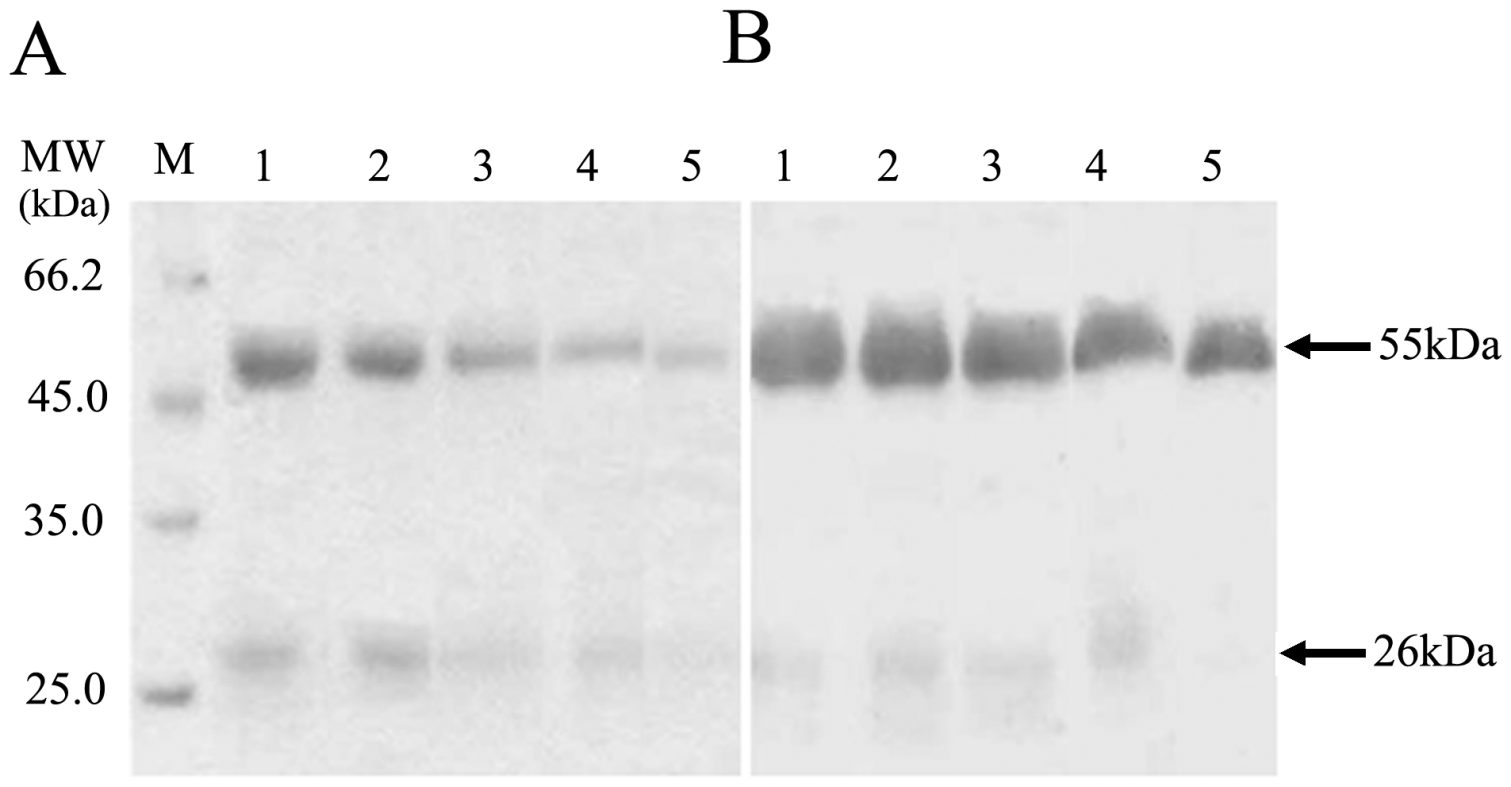
Detection of human IgG fractions purified by ion exchange and affinity chromatography. (A) SDS-PAGE and (B) immunoblotting analysis of different human IgG fractions. Rabbit anti-human IgG antisera (1∶1,000) and goat anti-rabbit IgG-HRP (1∶2,000) were used as the primary and secondary antibodies, respectively. M, molecular mass markers; 1, IgG; 2, unh-IgG; 3, unhs-IgG; 4, h-IgG; 5, hs-IgG.

Two IgG fractions, h-IgG and hs-IgG, were isolated from healthy human sera. The purity and apparent molecular weight of the proteins were analyzed by SDS-PAGE. Two bands at molecular weights of approximately 26 kDa and 55 kDa were observed for the two IgG fractions (Fig. 1A). The identity of these two bands was verified with rabbit anti-human IgG antibody (Fig. 1B). No other nonspecific bands was detected. As a control, the other three IgG species, namely, IgG, unh-IgG and unhs-IgG, were included in the analysis.

### Both IgG fractions possessed hemolytic activity

**Figure 2:**
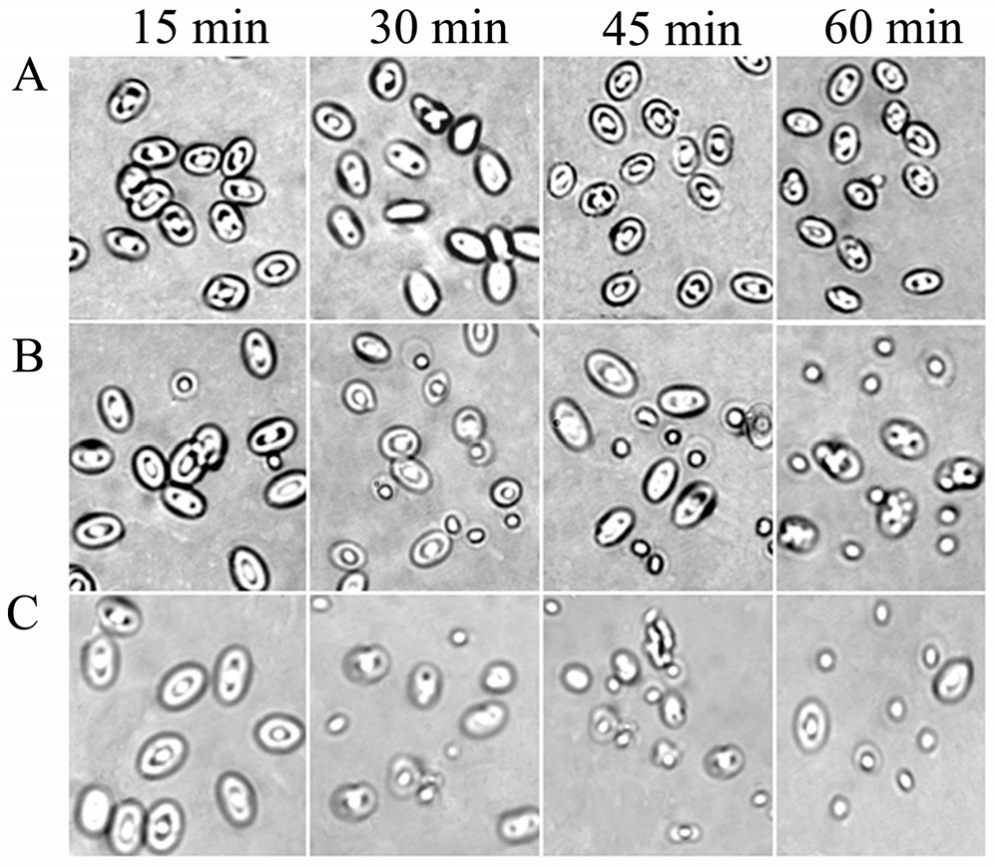
Kinetics of hemolysis of chicken erythrocytes by h-IgG. h-IgG was incubated with erythrocytes for 15, 30, 45 and 60 min at 37°C. The 0.5% (v/v) chicken erythrocyte suspensions were treated with (A) 0.01 M PBS, 10 mM CaCl_2_ (pH 6.0; negative control), (B) 5 µg/ml h-IgG and (C) ddH_2_O (positive control). The original digital images were magnified 172-fold.

Interestingly, the hemolysis assay showed that the two human IgG fractions (h-IgG and hs-IgG) could lyse human, rabbit, mouse and chicken erythrocytes within 1 h, and their hemolytic activities ranged from 5.29% to 100%, while hemolysis (except for type O human erythrocytes) by IgG, unh-IgG and unhs-IgG was almost negligible (Table 1). To determine the kinetics of the lysis reaction, chicken erythrocytes were incubated with h-IgG (5 µg/ml) for 15, 30, 45 and 60 min. After a 30-min exposure, some erythrocytes were lysed, and the extent of hemolysis increased with time until no cells were intact after the full 1-h incubation (Fig. 2). This confirmed that the h-IgG and hs-IgG fractions possessed hemolytic activity.

**Table 1:** Hemolysis of inhomogeneous erythrocytes by different human IgG fractions.

	Hemolytic activity (%) to inhomogeneous erythrocytes						
IgG Fractions	Human (A)	Human (B)	Human (O)	Human (AB)	Rabbit	Mouse	Chicken
IgG	0.00±0.01	1.46±0.03	5.77±0.05	0.60±0.01	0.00±0.00	1.12±0.01	0.00±0.00
unh-IgG	0.00±0.00	2.34±0.04	9.92±0.09	1.81±0.02	0.36±0.01	0.00±0.00	0.00±0.01
unhs-IgG	0.00±0.00	1.46±0.03	8.33±0.07	0.00±0.00	0.85±0.01	0.00±0.00	0.00±0.01
h-IgG	78.53±0.06**	70.04±0.08**	89.24±0.07**	75.90±0.07**	83.55±0.07**	93.89±0.06**	100.00±0.08**
hs-IgG	30.99±0.01**	34.99±0.06**	77.51±0.14**	62.68±0.05**	75.37±0.06**	5.29±0.01	57.97±0.10**

These data represent the mean ± SD of at least three separate experiments. We incubated 0.9 ml of 0.01 mg/ml IgG fractions with 0.3 ml of 0.5% (v/v) erythrocyte suspension at 37°C for 1 h.

** indicates a significant difference as compared to the control IgG (*P*<0.01).

### The IgG fractions might bind to erythrocyte membranes directly via a colloid osmotic pressure mechanism

**Figure 3:**
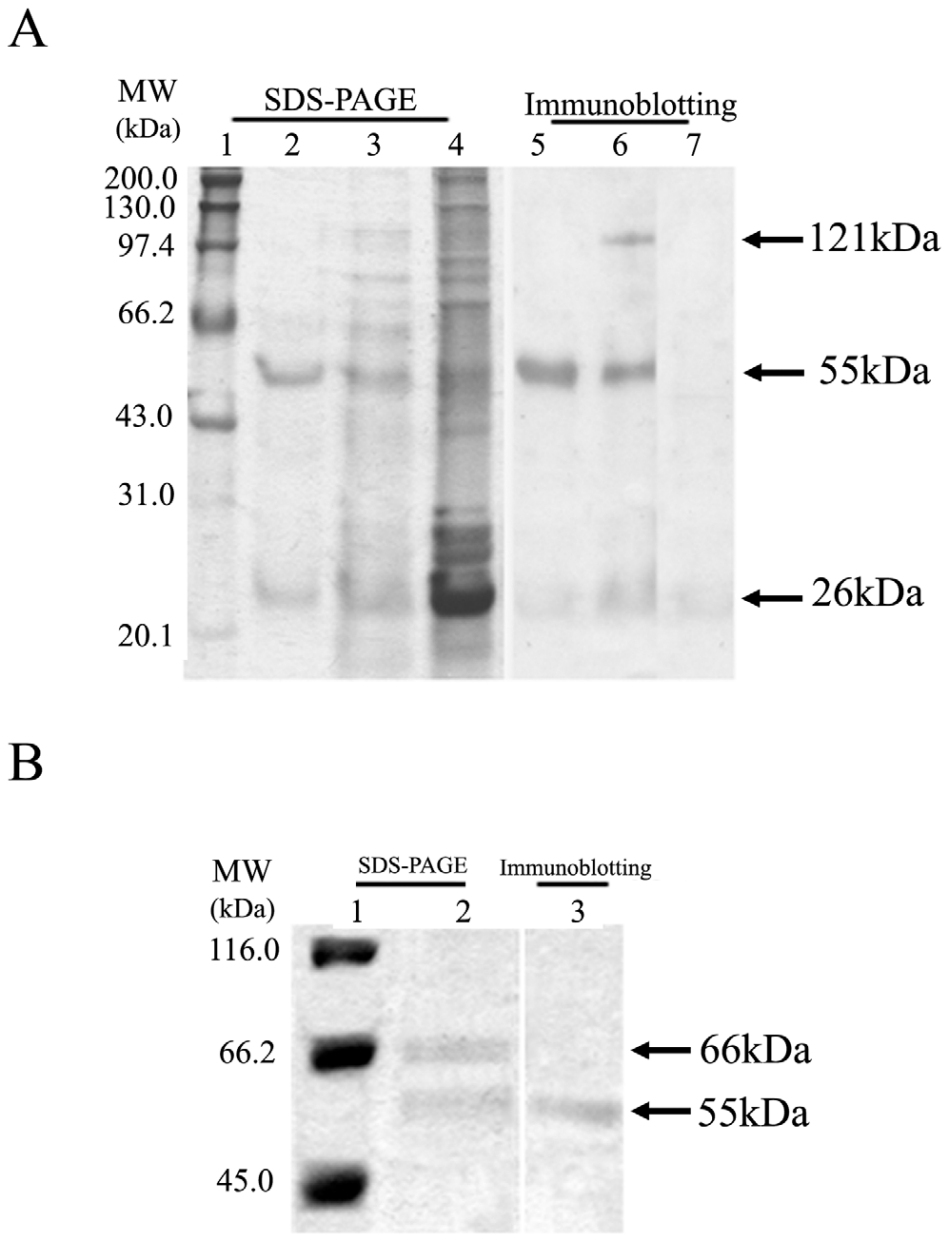
SDS-PAGE and immunoblotting of IgG fractions involved in hemolysis. Immunoblotting was performed using rabbit anti-human IgG antisera (1∶1,000) and goat anti-rabbit IgG-HRP (1∶2,000) as the primary and secondary antibodies, respectively. (A) analysis of proteins solubilized from the chicken erythrocyte membranes treated with h-IgG. 1, molecular mass markers; 2, h-IgG; 3 and 4, proteins from chicken erythrocyte membranes treated with h-IgG and double distilled water, respectively; 5–7, immunoblotting analysis of lanes 2–4. (B) analysis of the protein around 121 kDa following elution with electrophoresis. 1, molecular mass markers; 2, purified protein around 121 kDa; 3, immunoblotting analysis of lane 2.

To investigate whether the IgG fractions could bind to erythrocyte membranes directly, proteins solubilized from the hemolyzed chicken erythrocyte membranes were examined by SDS-PAGE and immunoblotting analysis. After immunobloting with anti-human IgG antibodies, 26-kDa and 55-kDa bands corresponding to the light and heavy chain of h-IgG, respectively, were observed in the erythrocyte membranes treated with h-IgG, but not with ddH_2_O (Fig. 3A). Importantly, a positive band about 121 kDa was also found (Fig. 3A). To further confirm the protein, it was cut out from the first SDS-PAGE gel and subjected to the second SDS-PAGE and immunoblotting analysis following elution with electrophoresis. Fig. 3B showed that the 121 kDa protein was separated into two bands of 66 and 55 kDa, and the 55 kDa band also could react with anti-human IgG antibody specifically, suggesting that the 121 kDa protein might be a compound of h-IgG heavy subunit and an erythrocyte membrane protein. Thus, it led to be deduced that the IgG fractions might bind to erythrocyte membrane directly.

**Figure 4:**
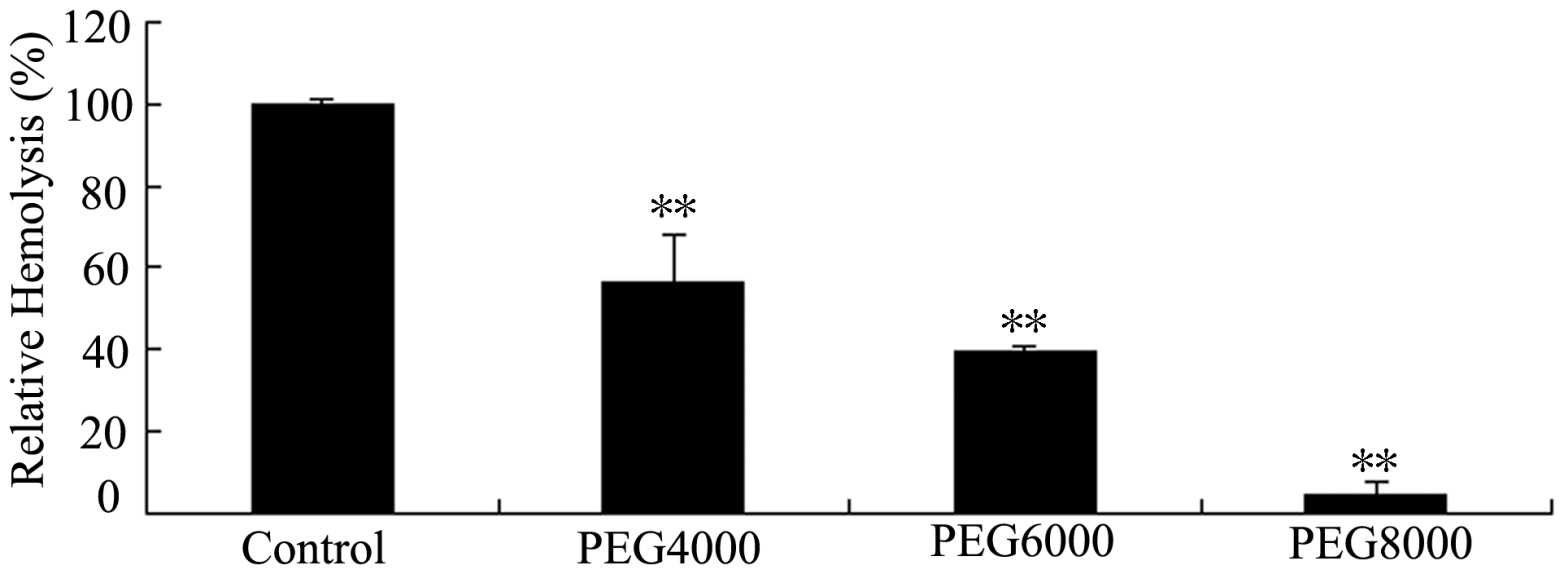
Osmotic protection against hemolysis by various osmoprotectants. 0.9-IgG (0.01 mg/ml) was incubated with 0.3 ml of 0.5% (v/v) chicken erythrocytes at 37°C for 1 h in the presence of various osmoprotectants. h-IgG incubated with chicken erythrocytes without osmoprotectant was used as a 100% hemolysis control. Data are expressed as means ± SD (n≥3). ** indicates a significant difference as compared to the control (*P*<0.01).

Further, the osmotic protection assay was performed to investigate the possible mechanism of the IgG fractions-induced hemolysis. When chicken erythrocytes were incubated with h-IgG in the presence of PEG 4000, 6000 and 8000, hemolysis was inhibited by 43.75±11.62%, 60.41±1.27% and 95.44±3.16%, respectively (Fig. 4). Nevertheless hemolysis was not completely inhibited by the osmoprotectants. The result suggested that erythrocytes were likely ruptured by colloid osmotic pressure shock after the IgG fractions had formed ion-permeable pores in the membranes.

### IgG protein polymorphism might be responsible for hemolytic activity

**Figure 5:**
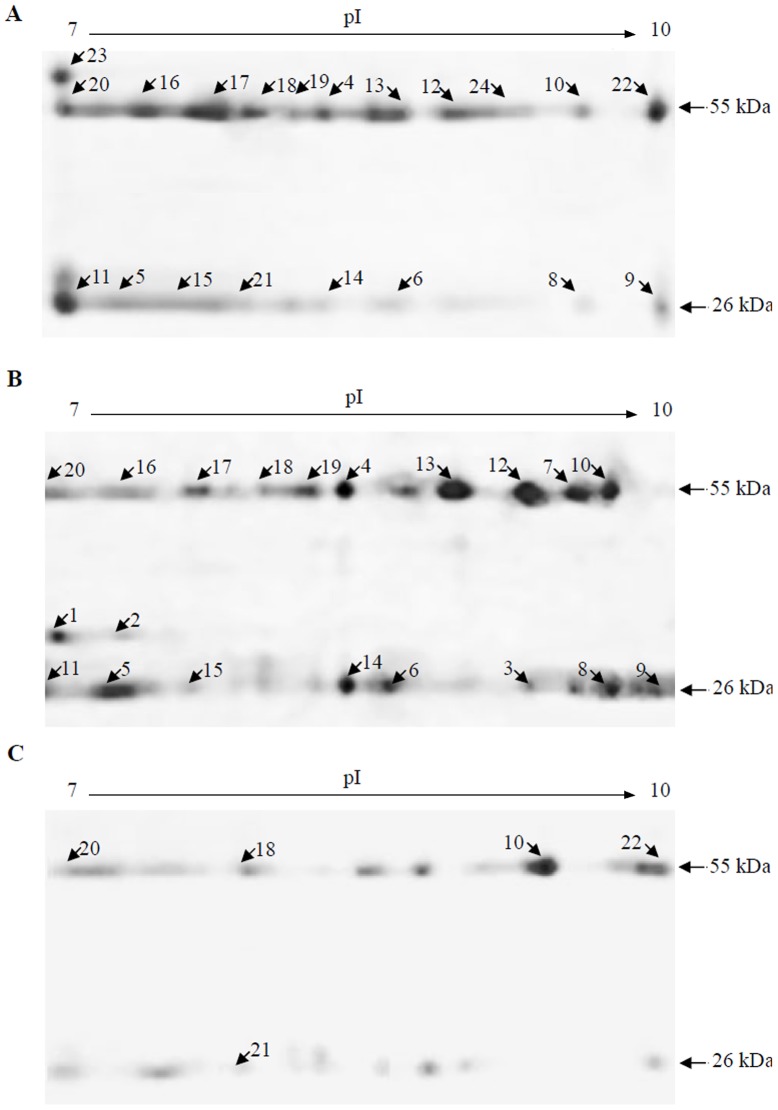
2-DE analysis of different human IgG fractions. The 2-DE gel images were analyzed with PDQuest software version 8.0 (Bio-Rad, CA). A, IgG; B, h-IgG; C, hs-IgG.

**Figure 6:**
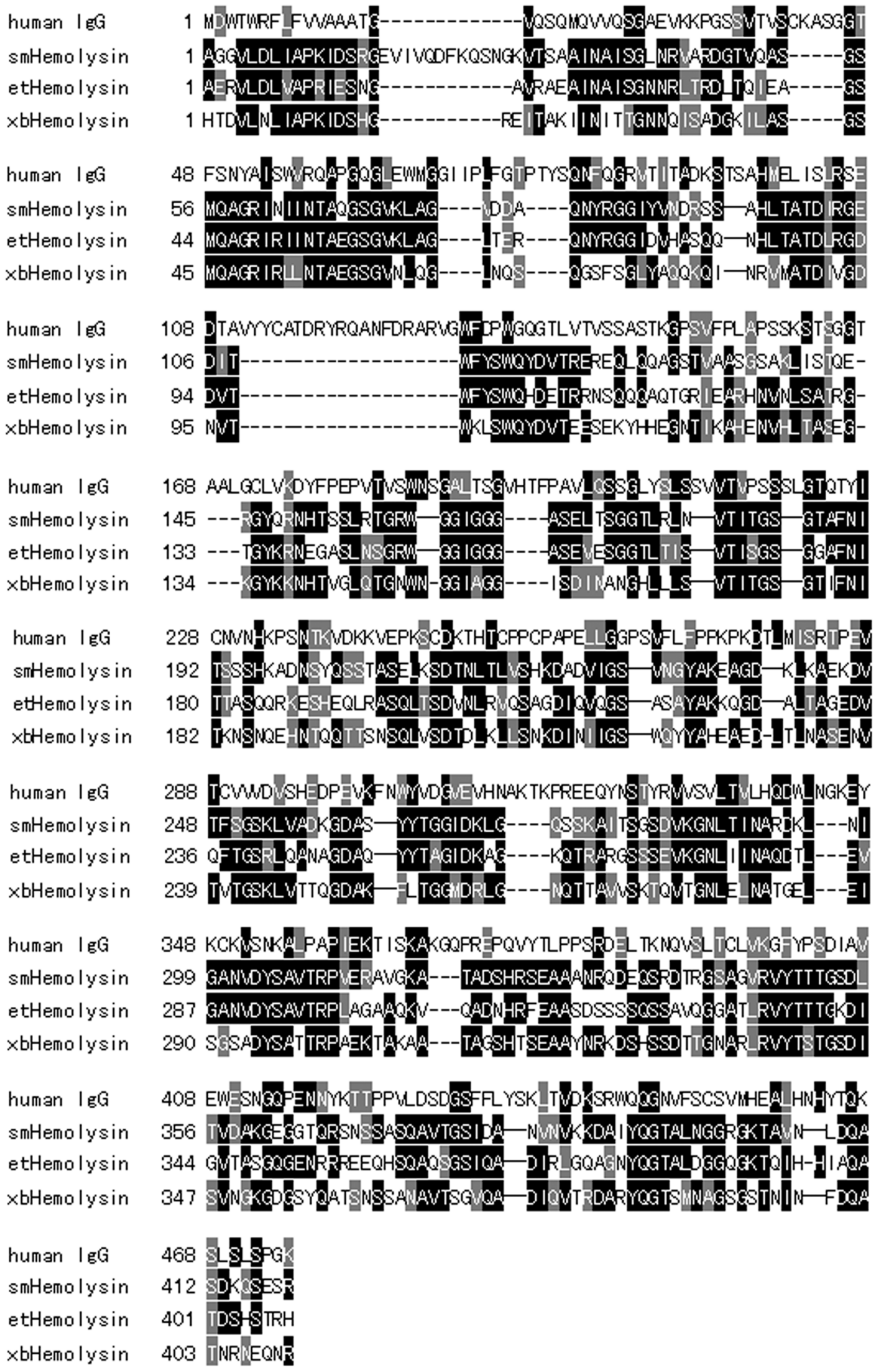
Multiple amino acid sequence alignments between human IgG and hemolysins. Black and gray show 100% and above 50% homology, respectively. GenBank accession numbers are: human IgG (AAA02914), hemolysin from *Serratia marcescens* (AAA50323), hemolysin from *Edwardsiella tarda* (ZP_06713740) and hemolysin from *Xenorhabdus bovienii* (YP_003466207).

To investigate the molecular basis for the hemolytic activity, comparative proteomics strategy was carried out. Following 2-DE, approximately 30 spots on each gel were distinguished by PDQuest software, mainly around 26 and 55 kDa. Twenty protein spots on the h-IgG gel were significantly altered compared with the IgG gel. Of these, spots 1–3 were present only on the h-IgG gel, spots 4–14 were upregulated, and spots 15–20 were downregulated (Figs. 5A and 5B). Similarly, a total of five significantly altered protein spots were observed in the hs-IgG gel. Of these, spot 10 was upregulated while spots 18, 20, 21 and 22 were downregulated (Figs. 5A and 5C). Regretfully, due to the highly diversity, some specific amino acid sequences related to the hemolytic properties in the specific IgG fractions have not been obtained by mass spectrometry (data not shown). Accordingly, an amino acid sequence alignment between human IgG and hemolysins was further produced. Figure 6 showed that homologies between human IgG heavy chain (AAA02914) and hemolysins from *Serratia marcescens*, *Edwardsiella tarda* and *Xenorhabdus bovienii* were about 28%, 24% and 20%, respectively. These findings imply that the protein polymorphism might be one of the molecular bases and responsible for hemolytic activity of IgG fractions, meanwhile, some potential specific sequences involved in the antigen nonspecific immunological function was also likely existed.

### The glycosylation diversity of the IgG fractions might also facilitate hemolytic property

**Figure 7:**
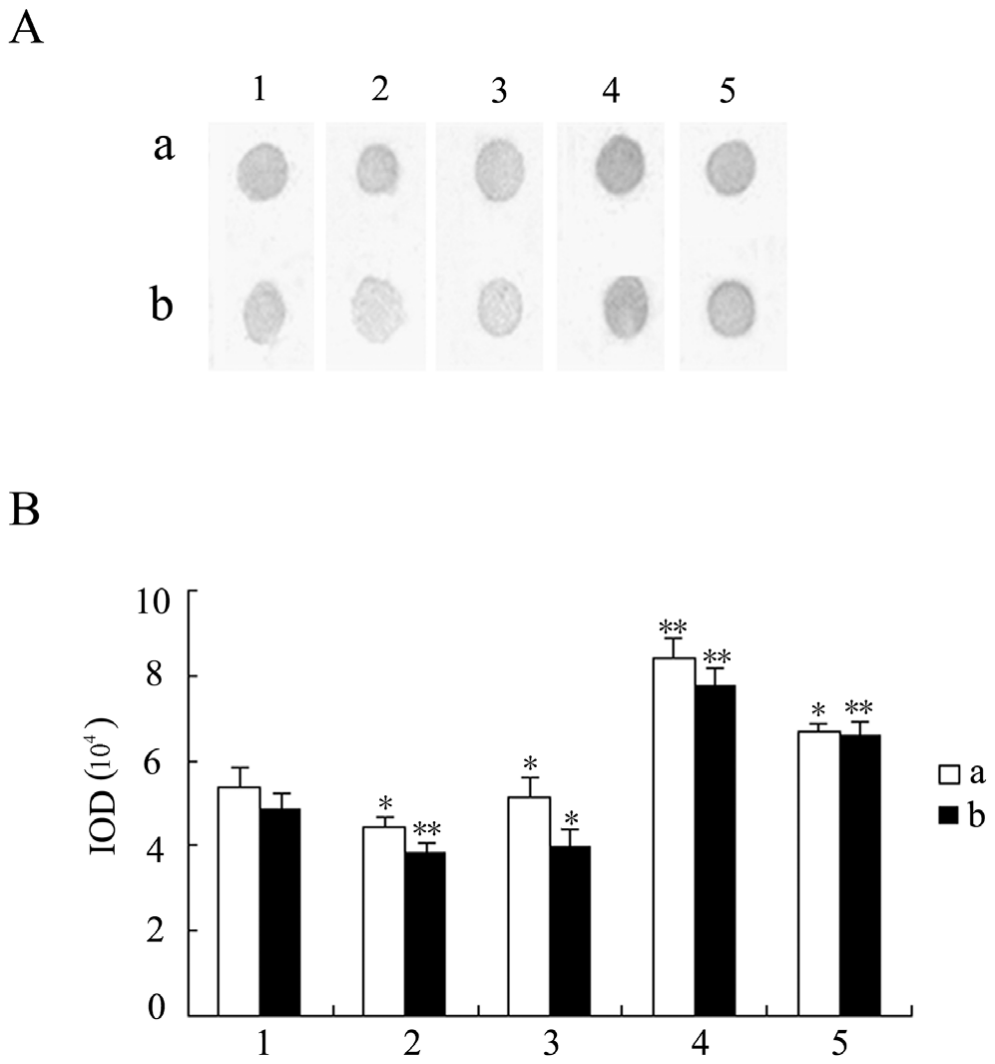
Analysis of the α-d-mannose/α-d-glucose content of different human IgG fractions by dot-blotting for lectin. Biotinylated ConA (1∶10) and avidin-peroxidase (1∶1,000) were used as the primary and secondary reagent, respectively. (A) dot-blotting for lectin. (B) bar graph showing the changes in spot intensity of human IgG fractions. Significant differences between the four different IgG fractions and the control IgG at each concentration were indicated with one (*P*<0.05) or two (*P*<0.01) asterisks. a and b, the concentration of the human IgG fractions was 0.3 and 0.15 mg/ml, respectively. 1, IgG; 2, unh-IgG; 3, unhs-IgG; 4, h-IgG; 5, hs-IgG.

Based on the above analysis, carbohydrate content analysis was further investigated. As shown in Table 2, the mean values for h-IgG and hs-IgG were approximately 3.6- and 2-fold higher than IgG, respectively. The α-d-mannose/α-d-glucose content of the IgG fractions was also different (Fig. 7A). All of the human IgG fractions could react specifically with ConA, and by measuring the intensity of the blotting, the α-d-mannose/α-d-glucose content was found to be in the order h-IgG>hs-IgG>IgG (Fig. 7B), which was in agreement with the carbohydrate content results. These evidences suggest that the diversity of IgG fraction in glycosylation level, like polymorphism in protein level, might also facilitate hemolytic property.

**Table 2:** Carbohydrate content of different human IgG fractions.

IgG Fractions	IgG	unh-IgG	unhs-IgG	h-IgG	hs-IgG
Carbohydrate content (µg/mg)	22.97±2.61	21.79±1.17	18.44±2.03	82.92±13.14**	47.58±3.32**

These data represent the mean ± SD of at least three separate experiments.

** indicates a significant difference as compared to the control IgG (*P*<0.01).

## Discussion

It was generally accepted that no close evolutionary relationship existed between the antigen nonspecific and antigen specific immunological molecules [28]–[29]. However, recent reports showed a significant association [30]–[32]. Antigen nonspecific proteins possess molecular polymorphism that exhibit primary adaptive immune functions [10]–[12]. At the same time, many antigen specific immunological molecules such as immunoglobulin share homologous domains or epitopes with antigen nonspecific immune factors [6]–[14]. This implies that human immunoglobulin may also share similar functions with antigen nonspecific immunological molecules.

In this study, we separated two human IgG fractions, h-IgG and hs-IgG, by affinity chromatography (Fig. 1) and characterized their hemolytic activity. h-IgG and hs-IgG showed obvious hemolytic activity against a variety of erythrocytes, while the control IgG, unhs-IgG and unh-IgG fractions did not (Table 1 and Fig. 2). This is somewhat in agreement with our previous findings that the agglutinative activity of *Litopenaeus vannamei* a-hemocyanin (purified by affinity chromatography) was increased 37-fold compared with that of *L. vannamei* s-hemocyanin (purified by size-exclusion chromatography) [14]. Furthermore, we found that the IgG fractions could bind to erythrocyte membranes directly (Fig. 3A). It is worth emphasizing that an approximately 121 kDa protein reacted with rabbit anti-human IgG antibody specifically was also found (Fig. 3A), then it was speculated as a compound of human IgG heavy chain and erythrocyte membrane protein based on the findings from the second SDS-PAGE and immunoblotting analysis (Fig. 3B). This result resemble those found for human IgG autoantibodies or Portuguese Man-of-War toxin binding to four or one erythrocyte membrane proteins in the hemolytic actions [33]–[34]. Besides, osmotic protection analysis indicated that the hemolysis mediated by h-IgG could be inhibited by PEG with different molecular weights in a degree (Fig. 4). These cumulative evidence implied that the IgG fractions might bind to erythrocyte membranes directly via a colloid osmotic pressure mechanism. This is similar to the results obtained for shrimp hemocyanin [19], sea anemone Equinatoxin II [35] and *Staphylococcus aureus* a-hemolysin [36] during hemolysis. However, direct evidence for the formation of ion-permeable pores is still missing, further investigations are required in the future.

To date, many hemolytic mechanisms have been found in human pathologic conditions [37]–[39]. For example, IgG and complement could mediate autoimmune hemolytic anemia [39]. However, the mechanism through which the erythrocyte meets its destruction in normal conditions remains controversial [40]–[42]. The present findings that two IgG fractions possessed nonspecific hemolytic properties against erythrocytes might provide an alternative approach to induce hemolysis in normal conditions in human. Although the exact physiological function of the hemolytic activity of the IgG fractions in vivo is yet to be determined, we believe this study provided strong evidences for the first time to associate nonspecific immune properties with human IgG, and offered an opportunity to learn more about the characterization of human IgG.

To investigate the origins of the h-IgG and hs-IgG hemolytic properties, a proteomic approach was employed. Significantly altered protein spots were found for h-IgG (20 spots) and hs-IgG (5 spots) compared to the control IgG (Fig. 5). The similar polymorphism of the IgG fractions to the nonspecific immune molecules of Toll-like receptor 4 (TLR4) [43], *Pacifastacus leniusculus* Dscam [44] and *Ciona* VCBPs [45], suggests that different isoforms are capable of recognizing and differentiating different pathogens. Regretfully, due to the highly diversity of IgG fractions, we failed to obtain some specific amino acid sequences related to the antigen nonspecific immune properties by proteomics analysis. Thereby, we decided to compare the amino acid sequence between human IgG and hemolysins using bioinformatics analysis. Interestingly, the results indicated that human IgG presented a significant degree of homology to three hemolysins from *Serratia marcescens*, *Edwardsiella tarda* and *Xenorhabdus bovienii* (Fig. 6). Collectively, these provides evidence that the protein polymorphism of human IgG fractions is likely to be responsible for the hemolytic property. At the same time, there might also exist some characteristic sequences involved in the antigen nonspecific immunological function in the IgG fractions, but further investigations are needed to identify the specific sequences.

To further investigate whether the hemolytic activity of IgG fractions was also related to its glycosylated modification, a glycomic strategy was performed. Interestingly, we found that both the carbohydrate and α-d-mannose/α-d-glucose content of h-IgG and hs-IgG were significantly higher (*P*<0.05) than that of control IgG (Table 2; Fig. 7), suggesting that higher glycosylation levels in IgG fractions might also contribute to the higher hemolytic activity. It led to be deduced that diversity in glycosylation level, as well as polymorphism in protein level, might together constitute the molecular bases of hemolytic properties of the human IgG fractions. Notably, although the h-IgG carbohydrate content was about 3.6-fold higher than that of IgG, no significant difference was observed between the unh-IgG and the control IgG (Table 2). This implied that only a subpopulation of the IgG fractions is likely to be involved in the hemolysis, the exact proportion is needed to further investigated.

Recent reports reveal that specific glycoforms of human IgG can be produced in different immune cells, and this is relevant to many physiological and pathological processes. In particular, recombinant IgG proteins produced in different host cells display different patterns of oligosaccharides [46]. Additionally, agalactosylated glycoforms of aggregated IgG may induce rheumatoid arthritis [47]. High mannosylation of surface IgM creates a functional bridge between human follicular lymphoma and microenvironmental lectins [48]. In this study, we demonstrated diversity in the carbohydrate and α-d-mannose/α-d-glucose content among IgG fractions. Furthermore, we also identified two protein spots (spot 1 and spot 2) that were present only in the h-IgG (Fig. 5) and had molecular weights that were higher than the light chains of IgG, indicating diversity in glycosylation. Unfortunately, although we tried to identify the specific glycans in h-IgG or hs-IgG for the relation to the hemolytic activity, no good data was obtained by mass spectrometry because of a limited carbohydrate content isolated from the specific IgG fractions. Thus, in the future, it will be interesting to investigate whether the oligosaccharides of human IgG fractions recognize pathogens nonspecifically and directly, and which oligosaccharides are involved in the hemolytic properties.

In summary, we show for the first time that human IgG fractions possess hemolytic activity, and identify polymorphisms in both the protein and oligosaccharide components that may be responsible for the antigen nonspecific immune properties. However, the identification of specific amino acid sequences or glycans of the IgG fractions involved in the function is left untested, further studies are required in the future. This work will assist future investigations into the evolutionary relationships between antigen nonspecific and antigen specific immunological molecules.
